# Time to Screen for Metabolic Dysfunction-Associated Steatotic Liver Disease (MASLD): Insights From a Single-Center Retrospective Risk Stratification Study in Saudi Arabia

**DOI:** 10.7759/cureus.108041

**Published:** 2026-04-30

**Authors:** Turky Zahrani, Al Hanouf Rayyani, Syed Anjum Gardezi

**Affiliations:** 1 Department of Family Medicine, Johns Hopkins Aramco Healthcare, Dhahran, SAU; 2 Department of Gastroenterology and Hepatology, Johns Hopkins Aramco Healthcare, Dhahran, SAU

**Keywords:** fatty liver disease, fibrosis, fibrosis-4 (fib-4) score, liver cirrhosis, liver function tests (lfts), metabolic dysfunction-associated steatotic liver disease, primary medical care

## Abstract

Background: Metabolic dysfunction-associated steatotic liver disease (MASLD) is the most common cause of chronic liver disease worldwide and is strongly associated with obesity and type 2 diabetes. However, many patients remain undiagnosed in primary care because liver enzyme levels are often normal despite the presence of clinically significant fibrosis. Current international guidelines recommend the use of noninvasive fibrosis scores, such as the fibrosis-4 (FIB-4) index, for risk stratification. Importantly, this study focuses on individuals with normal liver enzymes who are at risk of MASLD rather than those with an established diagnosis, positioning FIB-4 as a pragmatic screening tool to identify high-risk patients in primary care settings.

Objective: This study aimed to evaluate fibrosis risk among high-risk primary care patients with normal liver biochemistry and to assess referral patterns to gastroenterology services. This study provides the first real-world evidence from Saudi Arabia evaluating noninvasive fibrosis risk stratification in a primary care population with normal liver enzymes.

Methods: This retrospective observational study used electronic medical record data from primary care clinics within a large tertiary healthcare system in Saudi Arabia between January 2021 and December 2024. Adult patients (n = 200) with metabolic risk factors, including obesity and type 2 diabetes, with no previous diagnosis of liver disease and normal liver enzyme levels were included. FIB-4 scores were calculated using routinely available laboratory parameters to stratify patients into low, intermediate, and high fibrosis risk categories.

Results: A notable proportion of patients with normal liver enzymes demonstrated intermediate or high fibrosis risk based on FIB-4 scores. Referral rates to gastroenterology services were low, suggesting missed opportunities for early detection. Integrating FIB-4 into routine primary care pathways may improve early identification and referral of patients at risk of advanced fibrosis in MASLD.

Conclusion: These findings highlight the importance of proactive screening in high-risk individuals, even when liver enzymes are normal, as reliance on biochemistry alone may overlook clinically significant fibrosis; incorporating tools such as FIB-4 and the AST:ALT ratio can facilitate earlier identification and timely intervention.

## Introduction

Metabolic dysfunction-associated steatotic liver disease (MASLD), previously referred to as nonalcoholic fatty liver disease [1], has emerged as the most common chronic liver disease worldwide and represents a growing public health challenge. It is estimated that MASLD affects approximately one-third of the global population, with prevalence rates rising rapidly in regions with a high burden of metabolic risk factors such as obesity, type 2 diabetes mellitus, and dyslipidemia [2]. Countries in the Middle East, including Saudi Arabia, have witnessed a particularly sharp increase in metabolic syndrome and diabetes, placing a large proportion of the population at risk for MASLD and its complications [3]. Although most patients remain asymptomatic in the early stages, a subset may progress to steatohepatitis, advanced fibrosis, cirrhosis, and hepatocellular carcinoma, making early identification and risk stratification essential [4].

Primary care settings play a critical role in the early detection of MASLD because patients with metabolic risk factors are most frequently encountered in these clinics. However, the diagnosis of MASLD is often overlooked in routine practice [5]. One important reason is the reliance on liver biochemistry as a screening tool. A substantial proportion of patients with hepatic steatosis or even advanced fibrosis may have normal or only minimally elevated aminotransferase levels [6,7]. Consequently, normal liver enzymes can create a false sense of reassurance, leading to underrecognition of clinically significant liver disease. In many patients, fatty liver is detected incidentally during imaging studies performed for unrelated indications [8], while others may remain undiagnosed despite having multiple metabolic risk factors.

In recent years, international guidelines have emphasized the use of noninvasive fibrosis scores for the risk stratification of patients with suspected MASLD [9-11]. Simple and widely available tools such as the Fibrosis-4 (FIB-4) index [12] and aminotransferase ratios [13] can help identify individuals at increased risk of advanced fibrosis. These scores are particularly valuable in primary care because they rely on routinely available laboratory parameters and can be calculated easily without specialized equipment. Risk stratification using such noninvasive scores enables clinicians to categorize patients into low-, intermediate-, and high-risk groups, thereby facilitating more appropriate monitoring strategies and referral to specialist hepatology services when necessary.

Despite the availability of these tools, their integration into routine primary care practice remains inconsistent. As a result, many patients at increased risk of advanced fibrosis may not be referred for specialist evaluation in a timely manner [14]. Understanding the magnitude of this gap between risk identification and referral is essential for developing effective screening and referral pathways.

The present study had two objectives. The primary objective was to evaluate the burden of fibrosis risk among 200 primary care patients with normal liver biochemistry who were at risk of MASLD based on metabolic factors, incidental imaging findings, or noninvasive fibrosis assessments. The secondary objective was to assess referral patterns to gastroenterology services according to fibrosis risk stratification. By examining these patterns, this study highlights missed opportunities for early detection and informs the development of a practical risk-based referral algorithm that may help primary care physicians and nongastroenterology physicians identify patients who would benefit from specialist assessment, providing one of the first real-world evaluations of fibrosis risk stratification for MASLD in Saudi Arabia.

## Materials and methods

This retrospective observational study utilized electronic medical record data from primary care clinics within Johns Hopkins Aramco Healthcare, a large integrated healthcare system serving a diverse metabolic disease population, between January 2021 and December 2024.

Adult patients (≥18 years) attending primary care were identified using the electronic health record reporting tool (SlicerDicer, Epic Systems Corporation, Verona, WI) through structured queries based on metabolic risk factors and available laboratory data. Patients were screened for inclusion if they had features related to MASLD, including: 1) incidental hepatic steatosis detected on imaging performed for unrelated indications, 2) the presence of metabolic risk factors such as obesity, type 2 diabetes mellitus, dyslipidemia, or metabolic syndrome, and 3) availability of laboratory parameters required for noninvasive fibrosis assessment.

Only patients with liver biochemistry within the institutional reference range at the time of evaluation were included, defined as alanine transaminase (ALT) ≤50 IU/L and aspartate aminotransferase (AST) ≤59 IU/L based on local laboratory reference standards. Where multiple results were available, values closest to the index primary care encounter within a six-month window were used for analysis.

Patients with previously diagnosed chronic liver disease, including viral hepatitis, autoimmune liver disease, alcohol-related liver disease, or other secondary causes of steatosis, as well as those with established cirrhosis or prior gastroenterology follow-up, were excluded.

Demographic and clinical data were extracted from the electronic health record, including age, sex, body mass index (BMI), metabolic comorbidities, liver biochemistry, platelet count, and imaging findings when available. Cases with incomplete data required for FIB-4 calculation (n = 2) were excluded from the final analysis; no imputation was performed, given the minimal extent of missing data.

Noninvasive fibrosis assessment was performed with the FIB-4 index, calculated for all eligible patients from routinely available laboratory and demographic parameters. The FIB-4 score was derived as follows:

$\mathrm{FIB-4} = \frac{\text{Age (years)} \times \text{AST (U/L)}}{\text{Platelet count (10}^9\mathrm{/L)} \times \sqrt{\text{ALT (U/L)}}}$.

Based on established thresholds, patients were stratified into three fibrosis risk categories: low risk (FIB-4 <1.3), intermediate risk (FIB-4 1.3-2.67), and high risk (FIB-4 >2.67) for advanced fibrosis. In patients aged >65 years, a higher low-risk threshold of <2.0 was applied to improve specificity, in line with age-adjusted recommendations [15].

The primary outcome was the distribution of fibrosis risk categories among patients with suspected MASLD and normal liver enzymes in primary care. The secondary outcome was the proportion of patients in each risk category who were referred to gastroenterology services for specialist assessment. Referral was defined as a documented outpatient gastroenterology clinic visit or a formal electronic referral placed within 12 months of the index primary care encounter.

Descriptive statistics were used to summarize baseline characteristics and referral patterns. Continuous variables were reported as mean ± standard deviation or median (interquartile range), as appropriate, while categorical variables were expressed as frequencies and percentages.

The institutional review board approved the study, and all patient data were analyzed in a deidentified manner to ensure confidentiality.

## Results

A total of 200 patients met the study inclusion criteria. The cohort was predominantly female participants (n = 140, 70.0%) with a mean age of 58.7 years. All patients had obesity (n = 200, 100%; BMI ≥30 kg/m²) and type 2 diabetes mellitus (n = 200, 100%), representing a high metabolic risk population managed in primary care.

Using the FIB-4 index for fibrosis risk stratification, 123 patients (61.5%) were categorized as low risk, 71 patients (35.5%) as intermediate risk, and six patients (3.0%) as high risk for advanced fibrosis. Overall, 77 patients (38.5%) were classified as intermediate or high risk, indicating a substantial burden of potential advanced liver disease. Detailed demographic characteristics and fibrosis risk distribution are summarized in Tables 1, 2.

**Table 1:** Demographics, fibrosis risk distribution, and referral patterns Referral pattern refers to a documented gastroenterology/hepatology evaluation for MASLD or the absence of specialist consultation FIB-4: fibrosis-4; SD: standard deviation; MASLD: metabolic dysfunction-associated steatotic liver disease

Risk group	N	Age (mean ± SD)	FIB-4 (mean ± SD)	Female, n (%)	Male, n (%)	Referral pattern, n (%)
High	6	61.0 (±3.3)	3.12 (±0.48)	2 (33.3%)	4 (66.7%)	3 (50.0%) evaluated; 3 (50.0%) no referral
Intermediate	71	60.3 (±4.2)	1.61 (±0.29)	44 (62.0%)	27 (38.0%)	4 (5.6%) evaluated for MASLD; 41 (57.7%) had no referral; the remaining had an undocumented referral status
Low	123	57.7 (±6.2)	0.92 (±0.22)	94 (76.4%)	29 (23.6%)	Not applicable

**Table 2:** Clinical characteristics of patients with intermediate and high fibrosis risk All patients had normal LFTs at index evaluation AST: aspartate aminotransferase; ALT: alanine transaminase; LFTs: liver function tests

Parameter	Value, n (%) (n = 77)
Abnormal liver function tests	14 (18.2%)
AST:ALT ratio >1	56 (72.7%)
Imaging-confirmed hepatic steatosis	45 (58.4%)

Among patients classified as intermediate- or high-risk (n = 77), abnormal liver function tests were present in 14 (18.2%). An AST:ALT ratio >1 was observed in 56 patients (72.7%), and imaging-confirmed hepatic steatosis was documented in 45 patients (58.4%). These findings suggest that a significant proportion of patients at increased fibrosis risk may have normal liver enzyme levels, potentially leading to under-recognition of clinically significant disease.

Referral patterns to gastroenterology services varied across fibrosis risk categories. Among intermediate-risk patients, four patients (5.6%) were evaluated specifically for MASLD, while 41 patients (57.7%) had no documented specialty consultation. In the high-risk group, three patients (50.0%) were evaluated by gastroenterology, whereas the remaining three patients (50.0%) had no documented specialist assessment. These findings highlight a gap between fibrosis risk identification and specialist referral in primary care.

## Discussion

In this retrospective study evaluating patients with obesity and type 2 diabetes managed in frontline clinical settings, more than one-third of individuals were classified as having intermediate or high risk of advanced fibrosis based on the FIB-4 index. These findings highlight the substantial burden of potentially unrecognized liver fibrosis among patients with metabolic risk factors. Importantly, most of these patients had no prior liver-related evaluation, and many demonstrated normal liver enzyme levels. Together, these observations reinforce the growing recognition that MASLD represents a major and often underdiagnosed global health challenge.

The prevalence of MASLD has increased dramatically over the past two decades, paralleling the global rise in obesity, diabetes, and metabolic syndrome. Epidemiological estimates suggest that MASLD affects nearly one-third of the adult population worldwide [16], making it the most common chronic liver disease globally. However, the clinical impact of MASLD is largely driven by the subset of patients who develop progressive fibrosis, which remains the strongest predictor of liver-related morbidity and mortality [17]. Identifying individuals at risk of advanced fibrosis, therefore, represents a critical step in preventing long-term complications such as cirrhosis, hepatocellular carcinoma, and liver-related death [18]. Our findings suggest that a significant proportion of high-risk metabolic patients may already harbor elevated fibrosis risk even while remaining clinically asymptomatic.

Risk stratification using noninvasive fibrosis scores has emerged as a cornerstone of contemporary MASLD management. Among these tools, the FIB-4 index is widely recommended as an initial screening test because it relies on readily available clinical parameters, including age, aminotransferase levels, and platelet count [19]. Multiple international hepatology guidelines advocate a stepwise strategy that begins with simple, noninvasive scores in nonspecialist settings to identify individuals who may require further evaluation [9,10]. In this context, our findings demonstrate the practical utility of FIB-4 in identifying patients with elevated fibrosis risk within routine clinical practice. The distribution of risk categories observed in this study, with a substantial proportion of patients falling into intermediate- or high-risk groups, supports the feasibility of applying FIB-4-based risk stratification as part of everyday metabolic disease management.

In addition to composite fibrosis scores, simple biochemical indicators may also provide early clues to underlying fibrosis risk. In our cohort, an AST-to-ALT ratio greater than 1 was observed in a large proportion of patients classified as intermediate or high risk according to FIB-4. Although the AST/ALT ratio alone cannot establish the presence of fibrosis, it represents a readily identifiable signal that may prompt clinicians to consider further risk assessment [20]. Because the ratio can be recognized immediately when reviewing standard liver function tests, it may be particularly useful in frontline clinical settings where formal fibrosis scoring tools are not routinely calculated. When interpreted in conjunction with noninvasive scores such as FIB-4, the AST/ALT ratio may provide an additional layer of clinical context, helping clinicians identify patients who may benefit from further evaluation.

One of the most important observations in our study was the apparent gap between fibrosis risk identification and specialist referral. Despite a meaningful proportion of patients being categorized as intermediate or high risk for advanced fibrosis, relatively few had undergone gastroenterology evaluation. This discrepancy suggests that a significant number of patients with potentially progressive liver disease may remain unrecognized in routine practice. Several factors may contribute to this referral gap.

First, MASLD often remains clinically silent until advanced stages [21], and many patients present with normal or minimally elevated liver enzymes. As demonstrated in our cohort, abnormal liver biochemistry was present in only a minority of at-risk individuals. Reliance on liver enzyme abnormalities alone may therefore lead to missed opportunities for early detection [22].

Second, awareness of noninvasive fibrosis risk-stratification tools may remain variable among nonspecialist clinicians. Patients with metabolic disease are frequently managed by general practitioners, internists, diabetologists, and other physicians whose primary focus may be cardiovascular or metabolic risk management rather than liver disease detection. Without structured screening pathways, early fibrosis risk may therefore go unrecognized [11]. Integrating automated calculation of noninvasive scores, such as FIB-4, into electronic health record systems could represent a practical solution to this challenge by enabling automatic risk stratification whenever routine laboratory tests are performed [23].

The implications of these findings are particularly relevant in regions experiencing a rapid increase in metabolic disease burden. In Saudi Arabia and the wider Middle East, the prevalence of obesity, type 2 diabetes, and metabolic syndrome has increased substantially over recent decades [24]. Epidemiological studies suggest that MASLD prevalence in the region may exceed global averages, reflecting the combined impact of lifestyle changes, urbanization, and demographic transitions [25]. At the same time, healthcare systems across the region are evolving, and structured primary care frameworks are not uniformly established in all settings. Many patients with metabolic disease receive care not only within formal primary care clinics but also through general practitioners, internists, and other nongastroenterology physicians. In such environments, simple and widely accessible tools such as the FIB-4 index and AST/ALT ratio may provide practical mechanisms for recognizing fibrosis risk across a broad range of clinical contexts.

**Figure 1:**
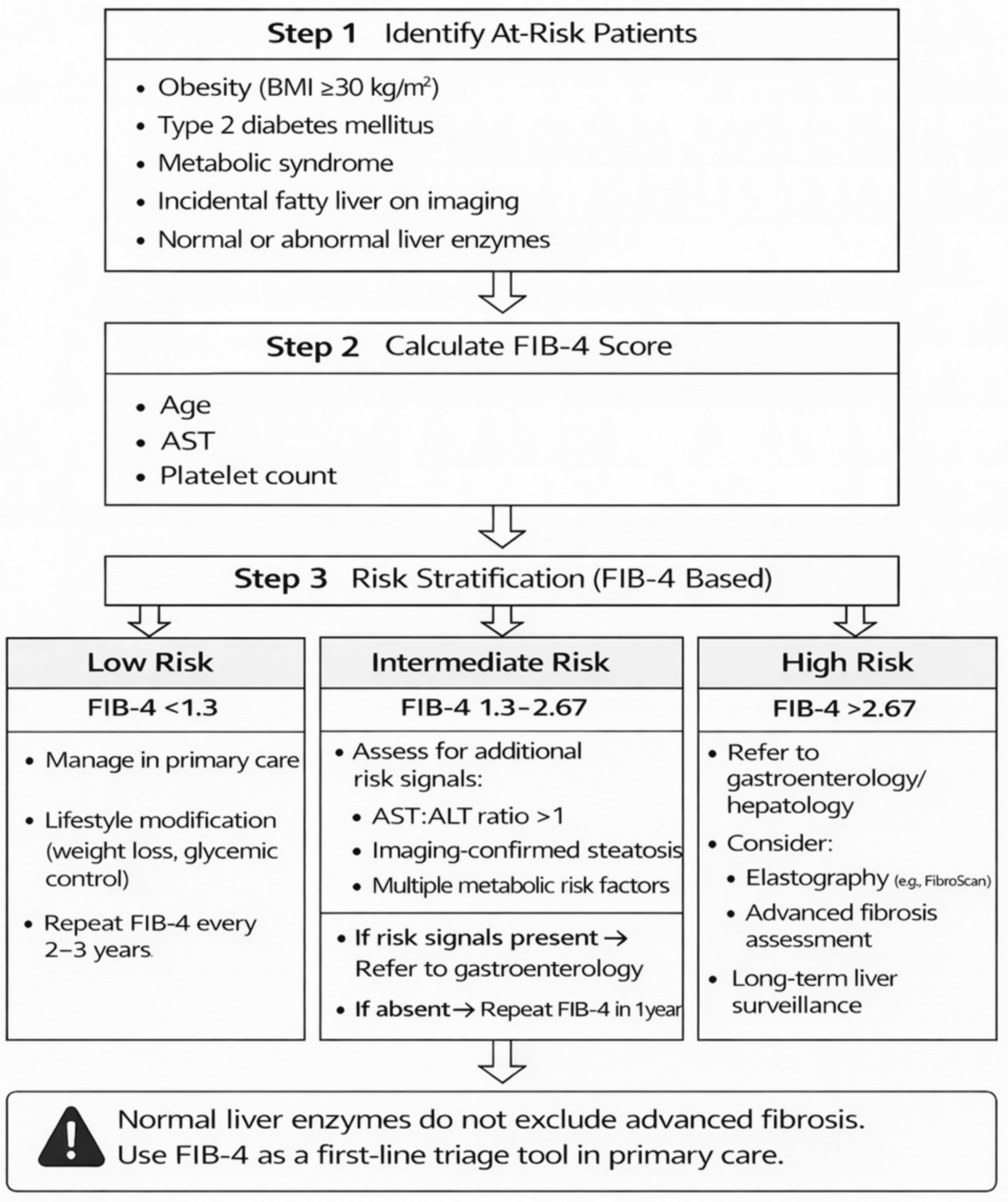
Primary care referral algorithm for MASLD risk stratification and specialist referral BMI: body mass index; FIB-4: fibrosis-4; AST: aspartate aminotransferase; ALT: alanine aminotransferase; MASLD: metabolic dysfunction-associated steatotic liver disease Image credit: This image was created by the author Syed Anjum Gardezi with Microsoft PowerPoint (Microsoft, Redmond, WA)

These findings contribute to the growing body of evidence suggesting that MASLD may represent a “silent fibrosis epidemic” developing within populations with metabolic disease [26]. Because many affected individuals remain asymptomatic and may demonstrate normal biochemical markers, significant fibrosis can remain undetected for years before clinical complications emerge. Early identification through simple risk-based strategies may, therefore, represent an important opportunity to intervene before irreversible liver damage occurs [27]. While universal population screening remains a subject of ongoing debate, targeted risk-based assessment in high-risk metabolic populations appears increasingly justified. A simple primary care referral pathway, as illustrated in Figure 1, provides a practical framework to facilitate early risk stratification and appropriate specialist referral in patients with MASLD. The pathway utilizes FIB-4 as an initial triage tool, enabling identification of low-risk patients for primary care follow-up while directing intermediate- and high-risk individuals toward further evaluation or specialist referral. Alongside improved risk stratification and referral pathways, emphasis on patient education and lifestyle modification, including weight reduction of 10%-15% of body weight, remains essential in the management of MASLD and should be integrated into primary care strategies.

Nevertheless, several limitations of this study should be acknowledged. The retrospective design introduces inherent constraints related to data completeness, potential misclassification, and selection bias. In addition, the study was conducted within a single healthcare system and included a relatively modest sample size, which may limit the generalizability of the findings to broader primary care populations. Fibrosis risk was assessed using a noninvasive score rather than confirmatory modalities such as elastography or histology; therefore, the results reflect risk stratification rather than a definitive diagnosis of advanced fibrosis. Furthermore, FIB-4 may be influenced by age, potentially leading to overestimation of fibrosis risk in older individuals despite the use of age-adjusted thresholds. Referral data were derived from electronic health records and may not fully capture external consultations or undocumented clinical decision-making.

Despite these limitations, the study provides important real-world insights into fibrosis risk detection within a high-risk metabolic population and highlights clinically meaningful gaps in current practice. By demonstrating that a substantial proportion of patients with obesity and type 2 diabetes may have elevated fibrosis risk despite frequently normal liver enzyme levels, these findings support the importance of simple, risk-based assessment strategies in frontline care. To our knowledge, this study represents one of the first analyses from Saudi Arabia evaluating fibrosis risk stratification using noninvasive scores in a primary care metabolic population, thereby providing regionally relevant data on the feasibility of implementing pragmatic screening approaches in routine clinical practice.

## Conclusions

In conclusion, our study demonstrates that a substantial proportion of patients with metabolic risk factors have an intermediate or high risk of advanced fibrosis based on noninvasive assessment, despite normal liver enzyme levels. Many of these individuals remain unrecognized and are not referred for specialist evaluation, highlighting important gaps in current primary care practice.

Routine incorporation of fibrosis risk stratification using simple tools such as the FIB-4 index, supported by readily available biochemical markers including the AST/ALT ratio, may facilitate earlier identification of at-risk patients and more appropriate referral pathways. In parallel, integrating patient education and preventive strategies, including lifestyle modification and weight reduction, remains essential in the management of MASLD.

While these findings reflect risk stratification rather than confirmed fibrosis, they underscore the potential value of pragmatic, scalable approaches in frontline care to address the growing burden of metabolic liver disease and reduce the long-term risk of liver-related complications.
